# Cytomegalovirus Infection in Inflammatory Bowel Disease Is Not Associated with Worsening of Intestinal Inflammatory Activity

**DOI:** 10.1371/journal.pone.0111574

**Published:** 2014-11-11

**Authors:** Alexandre Medeiros do Carmo, Fabiana Maria Santos, Carmen Lucia Ortiz-Agostinho, Iêda Nishitokukado, Cintia S. Frota, Flavia Ubeda Gomes, André Zonetti de Arruda Leite, Claudio Sérgio Pannuti, Lucy Santos Vilas Boas, Magaly Gemio Teixeira, Aytan Miranda Sipahi

**Affiliations:** 1 Departamento de Gastroenterologia, Hospital das Clínicas da Faculdade de Medicina da Universidade de São Paulo – LIM 07, São Paulo, São Paulo, Brazil; 2 Instituto de Medicina Tropical e Departamento de Doenças Infecciosas e Parasitarias (LIM-HC) da Faculdade de Medicina da Universidade de São Paulo, São Paulo, São Paulo, Brazil; 3 Instituto de Medicina Tropical e Hospital das Clínicas da Faculdade de Medicina (LIM-HC), Universidade de São Paulo, São Paulo, São Paulo, Brazil; 4 Departamento de Cirurgia do Serviço de Cirurgia do Cólon Reto e Ânus, Hospital das Clínicas da Faculdade de Medicina da Universidade de São Paulo, São Paulo, São Paulo, Brazil; University of San Francisco, United States of America

## Abstract

**Background:**

Cytomegalovirus is highly prevalent virus and usually occurs in immunocompromised patients. The pathophysiology and treatment of inflammatory bowel disease often induce a state of immunosuppression. Because this, there are still doubts and controversies about the relationship between inflammatory bowel disease and cytomegalovirus.

**Aim:**

Evaluate the frequency of cytomegalovirus in patients with inflammatory bowel disease and identify correlations.

**Methods:**

Patients with inflammatory bowel disease underwent an interview, review of records and collection of blood and fecal samples. The search for cytomegalovirus was performed by IgG and IgM blood serology, by real-time PCR in the blood and by qualitative PCR in feces. Results were correlated with red blood cell levels, C-reactive protein levels, erythrocyte sedimentation rates and fecal calprotectin levels for each patient.

**Results:**

Among the 400 eligible patients, 249 had Crohn's disease, and 151 had ulcerative colitis. In the group of Crohn's disease, 67 of the patients had moderate or severe disease, but 126 patients presented with active disease, based on the evaluation of the fecal calprotectin. In patients with ulcerative colitis, only 21 patients had moderate disease, but 76 patients presented with active disease, based on the evaluation of the fecal calprotectin. A large majority of patients had positive CMV IgG. Overall, 10 patients had positive CMV IgM, and 9 patients had a positive qualitative detection of CMV DNA by PCR in the feces. All 400 patients returned negative results after the quantitative detection of CMV DNA in blood by real-time PCR. Analyzing the 19 patients with active infections, we only found that such an association occurred with the use of combined therapy (anti-TNF-alpha + azathioprine)

**Conclusion:**

The findings show that latent cytomegalovirus infections are frequent and active cytomegalovirus infection is rare. We did not find any association between an active infection of CMV and inflammatory bowel disease activity.

## Introduction

Cytomegalovirus (CMV) is a ß herpes-virus with double-stranded DNA and represents a common viral infection in humans, with infection levels ranging from 40% in developed countries to 100% in developing countries [1]. Primary CMV infection is subclinical in most patients and is often followed by CMV disease or latent CMV infection. CMV disease is rare in immunocompetent patients but not among the immunocompromised population. The organs affected by CMV disease include the eyes, lungs, liver, urinary tract, pancreas, central nervous system, heart and gastrointestinal tract [2].

The different grades of CMV infection can be defined as not infected, latent infection, active infection and disease [3] (Table 1).

**Table 1:** The different grades of CMV infection.

CMV infection status	Description
**Not infected**	Not infected with the virus; negative IgG and IgM antibodies against CMV
**Latent infection**	Carrier of the CMV genome without active replication
**Active infection**	Detectable viral replication in peripheral blood or organs or a significant rise in IgM antibodies against CMV
**Disease**	Clinical expression of active infection, that is, active CMV infection with end-organ involvement

Inflammatory Bowel diseases (IBD), which include ulcerative colitis (UC) and Crohn's disease (CD), consist of chronic, non-specific inflammatory diseases of unknown etiology that affect the digestive tract and can be differentiated mainly due to the depth and extent of disease involvement. To determine the degree of inflammation, it is necessary to use a combination of parameter settings, covering clinical, endoscopic, laboratory, endoscopic and radiological values. The CDAI (Crohn's disease activity index) and the TrueLove-Witts are clinical indices that are widely used as a means to quantify the inflammatory activity of CD and UC, respectively [4], [5], [6]. In addition, Fecal Calprotectin (FC) is a very good marker of intestinal inflammation that has a close correlation with endoscopic and histological findings, as well as good sensitivity and specificity [7], [8].

A clinical treatment is not curative and often relies on the use of immunosuppressive drugs. The pathophysiology of a disease may compromise the immune system. This observation, in combination with the fact that CMV presents a tropism for sites of inflammation, prompted us to hypothesize whether patients with IBD are more susceptible to CMV infection and disease [9], [10].

Several authors have tried to correlate CMV and IBD, hypothesizing the following: possible triggering of CMV infection at the onset of IBD [11], [12]; a worsening of the infection [13], [14], [15], [16]; an association between the use of anti TNF-alpha and a higher risk of CMV disease [17], [18]; or the higher prevalence of CMV disease in UC patients, compared with CD [19], [20]. On the other hand, other authors have advocated that CMV is only a bystander or surrogate marker of severe colitis [21], [22], [23], [24], [25] or that the anti TNF-alpha therapies may reduce the risk of CMV reactivation in UC patients [26].

The prevalence of CMV infection in patients with IBD is highly variable. One reason for this wide variation is the difficulty and lack of standardization among indices for diagnosing the different grades of CMV infection [13], [27]. The prevalence of CMV infection and its clinical significance in IBD, especially in outpatients, have yet to be determined.

In this context, the aim of this study was to explore the prevalence of CMV infection in 400 outpatients with IBD, using serology, a real-time quantitative PCR assay on blood samples, and a qualitative PCR assay on fecal samples. Another objective was to correlate CMV viral replication with various demographic, therapeutic and inflammatory activities in IBD patients.

## Patients and Methods

### Patients and Samples

The study was carried out from June 2011 to August 2012 with IBD outpatients from the *Hospital das Clínicas*, School of Medicine, University of São Paulo (HC-FMUSP). The authors followed the Resolution number 196/96, the National Health Council/MS, respecting the basic principles of bioethics, autonomy, non-maleficence, beneficence and justice. The study was submitted to the Ethics Committee of the *Hospital das Clínicas*, Faculty of Medicine, University of São Paulo (USP-HC) (Research Protocol: 0144/11) and accepted in session on 11/05/2011, before it starts. All patients or guardians, after reading, filled in and signed the consent form and agreed to participate in the study. The diagnosis of inflammatory bowel disease, UC or CD, had been previously established by clinical, laboratory, endoscopic, radiological and histological findings, according to standard criteria.

Patients were asked to bring a fecal sample and were submitted to an interview on the day of consultation. Patients were evaluated to verify the levels of their disease activity, according to the pathology: CD (CDAI- Crohn's disease activity index) [5] and UC (Truelove-Witts index) [6]. After data collection, all patients were referred to the laboratory for collection of venous blood samples.

All of the data collected were analyzed and compared with data collected from these patients medical records. Those who did not meet the established criteria, did not sign the consent form, or failed to collect or deliver laboratory fecal materials were automatically excluded from the study.

### Definition of active CMV infection

Active CMV infection was defined by a positive IgM test and/or detection of CMV DNA in peripheral blood or fecal samples by qualitative or real time PCR

### Laboratory Analysis

Peripheral blood samples were collected, and the tests performed included the following: complete blood count, erythrocyte sedimentation rate, C-reactive protein(CRP), and IgG and IgM antibodies against CMV.

### Real Time Quantitative PCR (qPCR)

Extraction of viral DNA was performed by processing whole bloodusing a QIAamp DNA Mini-kit (Qiagen, Valencia, Calif.) according to the manufacturer's instructions.

For Amplification and detection of viral DNA, the sequences of the PCR primers and probe were selected from the US17 region of CMV AD169, as previously described [28]. Summarizing, forward and reverse primers were 5′-GCGTGCTTTTTAGCCTCTGCA-3′ and 5′-AAAAGTTTGTGCCCCAACGGTA-3′, respectively, and the probe FAM -TGATCGGCGTTATCGCGTTCTTGATC - BHQ. PCR was performed using TaqMan Universal PCR master mix (Applied Biosystems), prepared as follows: 20µL of DNA from whole blood was mixed with 25 µl of PCR master mix, 15 pmol of each of the primers, 10 pmol of the TaqMan probe, and DPOC water were added to final volume of 50 µl.

Reactions were performed using the ABI PRISM 7300 Real Time PCR System (Applied Biosystems, Foster City, Calif.) with the following cycling conditions: 1 cycle at 50°C for 2 min and 95°C for 10 min to activate the Taq-Polymerase followed by 45 cycles at 95°C for 15 s and 61°C for 1 min.

To determine the sensitivity of the method, standard DNA at a concentration of 15.000 copies/ml (DNA from ADN169, Sellex, Washington, DC, USA) was serially diluted until the concentration of 150 copies/ml and tested in triplicate.

For specificity, DNAs of the following pathogens: Epstein-Barr virus, Adenovirus, *Mycoplasma pneumoniae*, Herpes Simplex Virus Type 1, Herpes Simplex Virus Type 2, Varicella-Zoster Virus, *Pneumocystis jiroveci*, *Toxoplasma gondii*), obtained from commercial crops, acquired from SellexInc (Washington DC, USA), were subjected to PCR.

After performing the RT-PCR, DNA samples extracted from 300 of the 400 randomly selected patients) were analyzed by conventional qualitative PCR. The same qualitative technique was used for stool samples[29].

### Qualitative PCR

The fecal DNA was extracted using a Qiagen (QIAamp DNA stool Mini Kit) kit, and the detection of the cytomegalovirus genome was performed using the qualitative PCR technique (conventional) to amplify a specific segment of the CMV gB, with specific primers gB 1319 and gB 1604 of the glycoprotein B gene [29], [30].

The result was revealed by the presence or absence of a band at the expected segment of DNA electrophoresis on an agarose gel marked with ethidium bromide. Internal, negative and positive controls were performed in parallel to assess the proper operation of the reaction.

### Fecal Calprotectin

Stool samples were stored at −20°C to be thawed later, and a survey of fecal calprotectin using ELISA, PhiCal (Caprotectin Elisa Kit) was conducted [8], [31].

### Statistical Analysis

A statistical analysis to determine the characteristics associated with CMV and IBDs patients was conducted. Descriptive analyses were performed first, and the Fisher's exact test and Mann-Whitney test were used for categorical variables, with Student's t test being used for continuous variables. In a more detailed study, a simple and multiple logistic regression was considered. A P value <0.05 was considered significant.

## Results

### Clinical Characteristics

The clinical and demographic characteristics of the 400 patients involved in the study are shown in Table 2. Using clinical indices, including the Crohn's disease activity index (CDAI) for CD and the Truelove, for UC, only 67 CD patients and 21 UC patients presented with moderate or severe disease. However, using fecal calprotectin (FC), over half of the patients had an FC above 149 ng/ml, suggesting active inflammatory bowel disease. Analyzing these data, we found associations among FC≥150, CRP≥5 and anemia. The association between FC≥150 and the clinical indices (CDAI and Truelove) did not achieve statistical significance.

**Table 2:** Demographic and clinical profile of the inflammatory bowel disease patients.

Characteristics	Crohn's disease	Ulcerative Colitis
**Number of cases**	249	151
**Gender M:F**	113∶136	64∶87
**Age variation (mean)**	15–82 (45,5)	23–80 (49,92)
**Duration of disease, months (mean)**	12–456 (132,5)	6–576 (118,77)
**BMI, kg/m^2^ (mean)**	15,42–48,05 (24,3)	16,9–37,32 (25,51)
**Disease location**		
	**ileal** 64 (25,7%)	**distal** 50 (33,1%)
	**colonic** 65 (26,1%)	**left colonic** 41 (27,1%)
	**ileocolonic** 108 (43,3%)	**pancolonic** 60 (39,4%)
	**upper GI** 12 (4,8%)	
**Clinical Index (CDAI/Truelove)**		
Remission	126 (50,8%)	
Mild	55 (22,18%)	129 (86%)
Moderate	57 (22,98%)	21 (14%)
Severe	10 (4%)	0
**FC≥150, number (%)**	126 (50,6%)	76 (50,8%)
**Anemia, number (%)**	56 (22,5%)	28 (18,5%)
**C-reactive protein≥5, number (%)**	92 (37,7%)	56 (37,6%)
**Previous surgery**	100/247 (40,5%)	15/151 (9,9%)

The medications used are shown in Table 3. Immunosuppressive agents were widely used among these patients. Analyzing the combined therapy (anti TNF-α plus azathioprine), 37 patients with CD used infliximab and azathioprine, and 11 patients used adalimumab and azathioprine. Among UC patients, six used infliximab and azathioprine, and one used adalimumab and azathioprine.

**Table 3:** Medications used by the patients enrolled in the study.

Drug	Crohn's disease	Ulcerative colitis
Sulfasalazine	39 (15,7%)	70 (46,7%)
Mesalazine	55 (22,2%)	68 (45,3%)
Azathioprine	143 (57,7%)	41 (27,3%)
Steroids	23 (9,3%)	9 (6,0%)
Methotrexat	1 (0,4%)	0
Infliximab	66 (26,6%)	15 (10,0%)
Adalimumab	19 (7,7%)	2 (1,3%)
Ciprofloxacin	37 (14,9%)	10 (6,7%)
Metronidazole	23 (9,3%)	7 (4,7%)
Cyclosporine	0	1 (0,7%)
No medication	22 (8,9%)	13 (8,7%)
Combo therapy	48 (19.3%)	7 (4,7%)
**Total**	**248**	**150**

### CMV Status

The serologic status of CMV in the blood of patients is shown in Table 4. Only 24 patients were not infected with CMV, and 90,9% of patients exhibited IgG antibodies against CMV in the blood, indicating previous CMV infection. Ten patients were IgG positive, whereas 28 patients did not complete the tests. The analysis of fecal samples from 394 study patients by a qualitative PCR revealed that nine of them exhibited CMV DNA (Table 5). The detection of CMV DNA by RT-PCR in the blood produced values under the inferior limit (150 copies/mL) in all 400 patients. As a result of these overall negative results, we randomly selected 300 patients and repeated the detection of CMV by PCR in blood using qualitative PCR, the same technique used on the feces. Again, all of the samples were negative.

**Table 4:** Serologic distribution for CMV in patients with CD and UC.

Serologic	CD	UC	Total
**IgG (-) IgM (-)**	16 (7%)	8 (5,6%)	24 (6,5%)
**IgG (+) IgM (-)**	207 (90,4%)	131 (91,6%)	338 (90,9%)
**IgG (+) IgM (+)**	6 (2,6%)	4 (2,8%)	10 (2,6%)
**IgG (-) IgM (+)**	0	0	0
**Not performed**	20	8	28

**Table 5:** Analysis of CMV DNA by qualitative PCR on the feces of study patients.

Feces PCR	CD	UC	Total
**(+)**	5 (2,01%)	4 (2,65%)	9 (2,25%)
**(-)**	239 (95,98%)	146 (96,69%)	385 (96,25%)
**Not performed**	5 (2,01%)	1 (0,66%)	6 (1,5%)

The detection of CMV DNA by real time PCR in the blood produced values under the inferior limit (150 copies/mL) in all 400 patients. In order to double-check these overall negative results, we randomly selected 300 patients and repeated the detection of CMV in blood by using the same qualitative technique used on the feces. Again, all blood samples were negative.

Using serology and the results from the CMV DNA PCR, it is possible to conclude that 24 patients were not infected with CMV, 332 patients were infected without viral replication, and 19 were infected with viral replication. (Table 6)

**Table 6:** Patients distribution regarding CMV infection.

	CD	UC	Total
**Not infected**	16 (6.42%)	8 (5.3%)	24 (6%)
**Infected without viral replication (latent infection)**	204 (81.93%)	128 (84.77%)	332 (83%)
**Infected with viral replication (active infection)**	11 (4.42%)	8 (5.3%)	19 (4.75%)
**CMV IgM or feces PCR not performed**	18 (7.23%)	7 (4.63%)	25 (6.25%)

### Clinical-Virological Correlations

After dividing the patients into three different groups, the not infected, the latent infection and the active infection groups, we looked for associations between these groups and the variables of interest.

Analyzing exclusively CD patients, we found an association between the use of infliximab and CMV viral replication (p = 0.0045) and between the use of combined therapy and viral replication (p = 0.005). In UC patients, no variable was significantly associated with the three different groups of CMV infection.

Considering all patients with inflammatory bowel disease, the likelihood ratio test demonstrated a statistically significant association between the use of combined therapy and the CMV infection group (Table 7).

**Table 7:** Description of the cytomegalovirus groups, according to characteristics of interest in all patients and the results of the tests of association.

Variable	Not infected		Infected without replication		Infected with replication		Total	p	
	N	%	N	%	N	%			
Hemoglobin								0.195#	
**No anemia**	17	5.8	264	90.1	12	4.1	293		
**Anemia**	7	8.5	68	82.9	7	8.5	82		
CRP								0.395	
**<5**	14	6.1	207	90.0	9	3.9	230		
**≥5**	9	6.4	122	86.5	10	7.1	141		
Fecal calprotectin								0.464	
**<150**	10	5.6	163	90.6	7	3.9	180		
**≥150**	14	7.2	168	86.6	12	6.2	194		
Sulfasalazine								0.587	
**No**	19	7.0	237	87.5	15	5.5	271		
**Yes**	5	4.9	94	91.3	4	3.9	103		
Mesalazine								0.704	
**No**	18	6.9	230	88.5	12	4.6	260		
**Yes**	6	5.3	101	88.6	7	6.1	114		
Azathioprine								0.165	
**No**	10	5.1	181	91.4	7	3.5	198		
**Yes**	14	8.0	150	85.2	12	6.8	176		
Steroids								0.433#	
**No**	21	6.1	305	89.2	16	4.7	342		
**Yes**	3	9.4	26	81.3	3	9.4	32		
Ifx								0.068#	
**No**	22	7.3	266	88.7	12	4.0	300		
**Yes**	2	2.7	65	87.8	7	9.5	74		
Ada								0.066#	
**No**	24	6.8	314	88.7	16	4.5	354		
**Yes**	0	0.0	17	85.0	3	15.0	20		
Combo therapy								**0.001#**	
**No**	24	7.4	287	88.9	12	3.7	323		
**Yes**	0	0.0	44	86.3	7	13.7	51		

Result of the chi-square.

# Result of the likelihood ratio

Utilizing the variables in univariate tests with descriptive levels below 0.2 (p<0.2), we verified whether the infection's characteristics as whole could influence replication. We found that a patient using combined therapy had a chance of replication that was 3.63 times greater than that of patients not using this treatment (p = 0.014) (Table 8).

**Table 8:** Results of the multiple logistic regression model to explain the replication of cytomegalovirus.

Variable	OR	IC (95%)		P
		Inferior	Superior	
**Hemoglobin**				
**No anemia**	1.00			
**Anemia**	1.38	0.45	4.28	0.576
**CRP**				
**<5**	1.00			
**≥5**	1.54	0.54	4.38	0.419
**Combo therapy**				
**No**	1.00			
**Yes**	3.63	1.30	10.12	**0.014**
**Results of the logistic regression**				

## Discussion

This study examined patients with IBD. Without any previous selection, 400 outpatients were randomly selected in order to evaluate a group that would best represent the general population of patients with IBD from the HCFMUSP.

More than half of these patients had intestinal disease activity, as confirmed by fecal calprotectin. Based on the clinical index, most patients were categorized with quiescent/mild disease (50.8%/22.18%CD and 0/86% of UC). However, such clinical indices have been criticized due to their highly variable sensitivity and specificity, and we believe that fecal calprotectin is the better parameter to demonstrate that half of the patients had active IBD. These results corroborate the literature, which is quite critical of these indices but reports a good sensitivity and specificity of fecal calprotectin in relation to endoscopic findings [8], [31], [32].

The very high percentage of patients infected with CMV, as evidenced by the presence of IgG antibodies to CMV, can be justified by the socioeconomic status of the study group [19], [33], [34]. Using Fischer's test and simple logistic regression, we did not find significant differences between the group of uninfected patients and the group with latent infections in relation to the activity index of IBD, CRP levels or fecal calprotectin. This contradicts the original hypothesis, which held that the latent CMV infection could worsen the IBD activity compared to the uninfected CMV patients [14], [15], [16], [35]. It is worth noting that only 24 patients (6.45%) were not infected, instead having negative antibodies IgG and IgM for CMV.

Despite the large number of patients undergoing immunosuppressive therapy, comprising more than half of patients with active IBD, viral replication was not detected by PCR in their blood in any case. These null results may have been obtained because our sample was composed of IBD outpatients, rather than a specifically selected population. Our study corroborates other studies arguing that the frequency of viral replication and CMV disease in patients with IBD is not as high as sometimes reported, questioning the virulence of CMV in patients with IBD [21], [24], [25], [26], [27]. The detection of CMV DNA in the fecal specimens by a qualitative PCR assay employing primers to the gB region, a highly variable region of HCMV, could underestimate the number of patients shedding CMV in feces. However, as this assay was performed blindly in all samples regardless of the clinical category or activity index of inflammatory bowel disease, we believe that our result concerning the lack of association between CMV and worsening of intestinal inflammatory activity was not affected.

When we began our study, the association between IBD and CMV was controversial, with the literature pointing to the risk that the rates of CMV infection, replication and disease might be higher in IBD patients. As the present project has progressed, new studies have emerged to challenge these ideas, suggesting that CMV is an occasional finding, a bystander in IBD enterocolitis [21], [24], [25], [26], [27].

In our series of ulcerative colitis patients, we did not find any patients with a severe form of the disease, according to the Truelove-Witts classification, which affects the assessment of the role of CMV in this particular group of patients. CD patients were found in all categories of CMV infection, but we did not find any correlation between CMV and the severity of IBD. We found that, in patients with mild and moderate forms of CD and UC, there was no association between the severity of IBD and CMV viral replication in the blood or stool specimens.

Pillet et al. [26] suggest that immunosuppressive therapy does not appear to have an impact on CMV reactivation in CD patients. They state that TNF alpha enhances CMV viral replication and that anti-TNF drugs may reduce the risk of CMV reactivation. However, we detected CMV viral replication in UC and CD patients, indicating that anti-TNF alpha does not protect against viral replication. In CD patients, an association was found between the viral replication of CMV and anti-TNF alpha, contradicting the hypothesis put forth by Pillet et al.

In the present study, we found a statistically significant association between CMV viral replication and the use of combined therapy, consisting of biological therapy (infliximab or adalimumab) plus an immunosuppressive drug (azathioprine or methotrexat). We believe that higher immunosuppression favors viral replication. However, the increase in CMV replication was not accompanied by a greater intestinal inflammatory activity, as evidenced by fecal the calprotectin levels. The present findings further support the hypothesis that CMV is a spectator of the inflammatory process in IBD. The higher the degree of immunosuppressive state caused by the therapy and/or disease is, the greater the chance for CMV replication; however, this finding did not seem to worsen the IBD activity.

The methodological difficulties in achieving a clear categorization of the CMV infections in uninfected, infected non-replicating, and infected with replication and with CMV disease patients might jeopardize the critical evaluation of each work. This effect can be seen in the large variation among the rates of CMV disease that have been published in previous works, suggesting that many cases of the disease were most likely misdiagnosed as CMV infection or CMV replication.

Based on the improved standardization of diagnostic methods and increasing understanding of the pathophysiology of CMV, studies should soon converge on lower rates of diagnosis for CMV disease in the IBD population.

In summary, our study has shown that latent infection by CMV was quite prevalent in a study group of patients with inflammatory bowel disease. We found no association between IBD activity and CMV infection in IBD patients, even among patients with viral replication using immunosuppressive therapy. Finally, although the use of combined therapies in patients with IBD was associated with the viral replication of CMV, such therapies bore no relation to inflammatory activity of IBD.
