# Correction: Cytomegalovirus Infection in Inflammatory Bowel Disease Is Not Associated with Worsening of Intestinal Inflammatory Activity

**DOI:** 10.1371/journal.pone.0133102

**Published:** 2015-07-14

**Authors:** Alexandre Medeiros do Carmo, Fabiana Maria Santos, Carmen Lucia Ortiz-Agostinho, Iêda Nishitokukado, Cintia S. Frota, Flavia Ubeda Gomes, André Zonetti de Arruda Leite, Claudio Sérgio Pannuti, Lucy Santos Vilas Boas, Magaly Gemio Teixeira, Aytan Miranda Sipahi

There is a correction to the data availability statement as follows: Data are available upon request from the authors with approval from the Ethics Committee of the Hospital das Clínicas, Faculty of Medicine, University of São Paulo (USP-HC) (Research Protocol: 0144/11), due to confidentiality restrictions. Requests may be sent to Dr. Alexandre Medeiros do Carmo at amccirurgia@gmail.com.
